# Simple microfluidic devices for *in situ* detection of water contamination: a state-of-art review

**DOI:** 10.3389/fbioe.2024.1355768

**Published:** 2024-02-02

**Authors:** Buthaina A. AlMashrea, Ahmed M. Almehdi, Samar Damiati

**Affiliations:** ^1^ Department of Chemistry, College of Sciences, University of Sharjah, Sharjah, United Arab Emirates; ^2^ Chemical Analysis Laboratories Section, Dubai Central Laboratory Department, Dubai, United Arab Emirates

**Keywords:** micro-total analytical system, lab-on-a-chip, water contaminants, *in situ* detection, water quality monitoring, inorganic and organic pollutants, image processing

## Abstract

Water security is an important global issue that is pivotal in the pursuit of sustainable resources for future generations. It is a multifaceted concept that combines water availability with the quality of the water’s chemical, biological, and physical characteristics to ensure its suitability and safety. Water quality is a focal aspect of water security. Quality index data are determined and provided via laboratory testing using expensive instrumentation with high maintenance costs and expertise. Due to increased practices in this sector that can compromise water quality, innovative technologies such as microfluidics are necessary to accelerate the timeline of test procedures. Microfluidic technology demonstrates sophisticated functionality in various applications due to the chip’s miniaturization system that can control the movement of fluids in tiny amounts and be used for onsite testing when integrated with smart applications. This review aims to highlight the basics of microfluidic technology starting from the component system to the properties of the chip’s fabricated materials. The published research on developing microfluidic sensor devices for monitoring chemical and biological contaminants in water is summarized to understand the obstacles and challenges and explore future opportunities for advancement in water quality monitoring.

## 1 Introduction

Water security and sustainability have become a concerning issue for many governments due to the rapid increase in population growth, urbanization, climate change, and industrial activities affecting the continuous supply of clean water. According to the WHO 2023 article concerning the 2 billion people living in water-deficient countries, 296 million use water from unsafe wells, and 115 million collect untreated water (World Health Organization, 2023). The UN 2022 report shows that 3 billion people use water bodies without quality monitoring (Nations, 2022). These natural water bodies can be contaminated from point sources, such as industrial and domestic discarded waste, or non-point sources, such as runoff that drains the waste from agriculture and contaminated soils into rivers and lakes (Rai et al., 2022). The main challenge is finding alternative water sources that can be used for daily human activities and agriculture to avoid the depletion of natural water resources. The WHO states that reducing water depletion by reusing wastewater is an important strategy. However, performing this practice randomly without regulation and monitoring will pose risks to the ecosystem and human health (World Health Organization, 2023). Developing a rapid method for monitoring water quality is also necessary.

Monitoring water pollutants is crucial in this situation, and lab testing plays a core role in providing data to decision-makers to carry out treatment processing and wastewater reuse. Water pollutants can be divided into three categories: inorganic pollutants, such as nutrients and heavy metals; organic pollutants, such as chemical oxygen demand (COD); and biological pollutants, for instance, *Escherichia coli*, *salmonella*, and other bacteria. The traditional methods for monitoring water pollutants use heavy instrumentation, such as inductively coupled plasma (ICP), ion chromatography (IC), gas chromatography (GC), and UV-VIS spectrophotometers, which provide high-efficiency quantitative and qualitative results. Nevertheless, they are unsuitable for onsite detection, requiring expensive maintenance and highly skilled operators. The technological revolution has provided scientists with techniques that can mimic the efficiency and sensitivity of traditional detection methods.

A research hot spot of lab technology is microfluidics, also known as “lab-on-a-chip” (LOC) devices or micro-total analytical systems (μ-TAS), which are microdevices that contain microchannels and microchambers where the fluid flows through holes in dimensions ranging from tens to hundreds of micrometers (Whitesides, 2006). In 1990, Manz and co-workers were the first to develop the microfluidic chip concept, a miniaturized total chemical analysis system that can be used for ecosystem monitoring (Manz et al., 1990; Schulze et al., 2017). In 2004, the *Business 2.0* magazine journal chose LOC as “One of the seven new technologies that will change everything” in the cover article (Zachary et al., 2004). These miniaturized devices can functionalize the chemical reaction in a small platform chip, representing many advantages for *in situ* detection, such as acting as portable sensors, decreasing the consumption amounts of samples and reagents, and reducing the reaction time (Nge et al., 2013). Novel chip materials include glass, paper, and polymers. There are diverse detection methods, for instance, electrochemical and optical methods (Alhalaili et al., 2022). Therefore, depending on the application that the microfluidic chip will be used for, multiple factors should be considered when fabricating LOC devices, such as the type of chip and the compatibility of the chip’s material and dimensions with the reagent of the analyte that will be detected (Damiati et al., 2018a; Damiati et al., 2020; Damiati et al., 2022a; Damiati et al., 2022b). This technology is mainly applied for genetic diagnoses and biological applications. Many researchers have investigated the routes for applying this technology to monitor water pollutants such as nutrients, heavy metals, and biological and organic pollutants.

**FIGURE 1:**
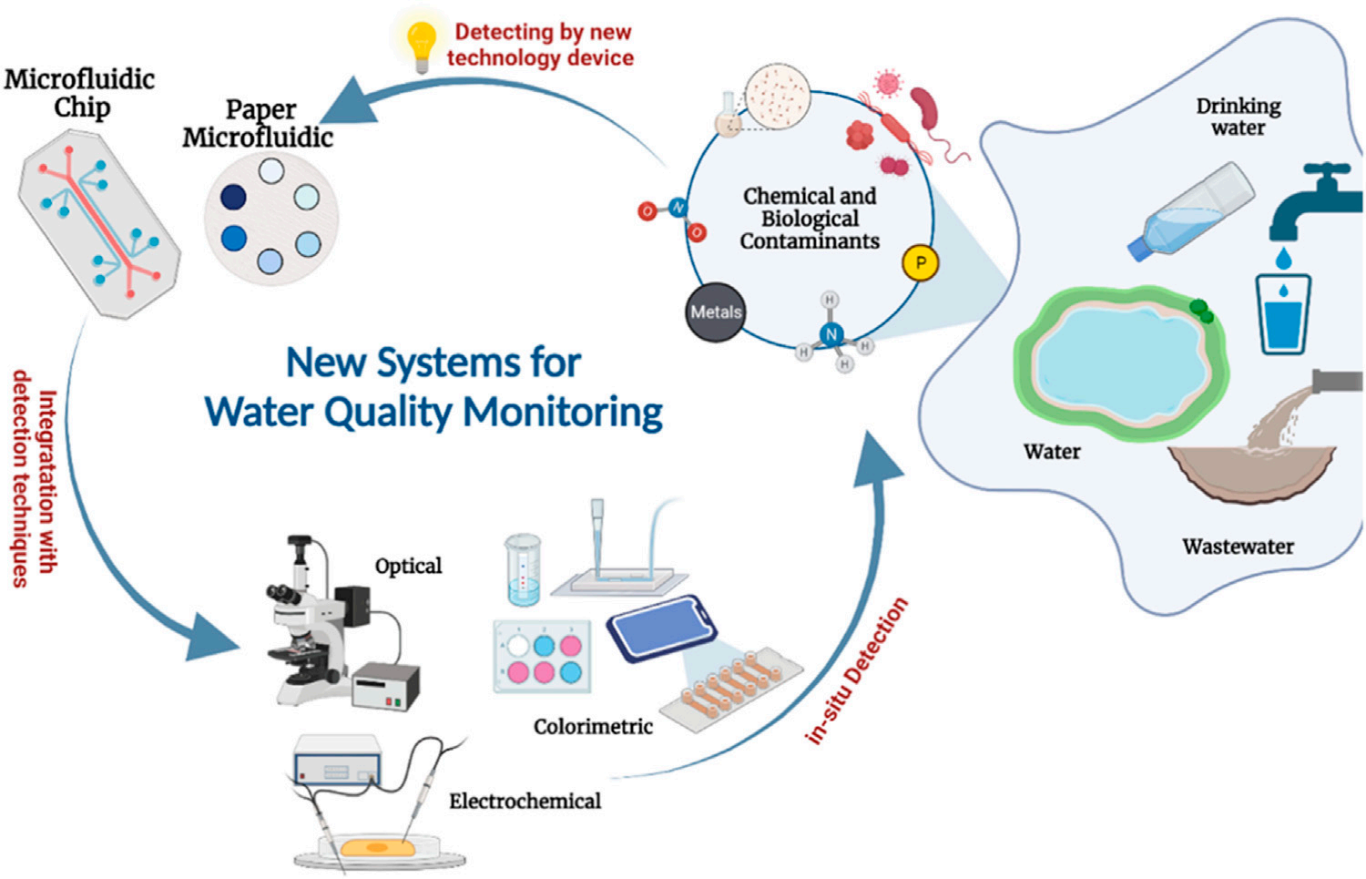
Schematic representation of microfluidics applications integrated with different techniques for the detection of common contaminants in water [Created with BioRender.com].

This review summarizes the sophisticated system features of microfluidic technology devices for monitoring water pollutants (Figure 1). Initially, we discuss the basics of microfluidic technology, from the system components to the properties of materials that can be used to fabricate the chips. Then, we summarize various research applications concerning the fabrication and development of microfluidic devices for monitoring water contaminants. Finally, we highlight the challenges of LOC devices and explore the possibilities of their enhancement.

## 2 Basics of microfluidic technology

The principle behind microfluidics is to control the movements of fluids at the microscale in a chip consisting of many micro-sized system components to miniaturize chemical reactions using a small volume of samples and reagents, which also can be integrated with different detection methods. The chip can be constructed from a variety of material types based on their high compatibility with the analyte target to help achieve precise control of the behaviors of the fluids.

### 2.1 System components and detection methods

**FIGURE 2:**
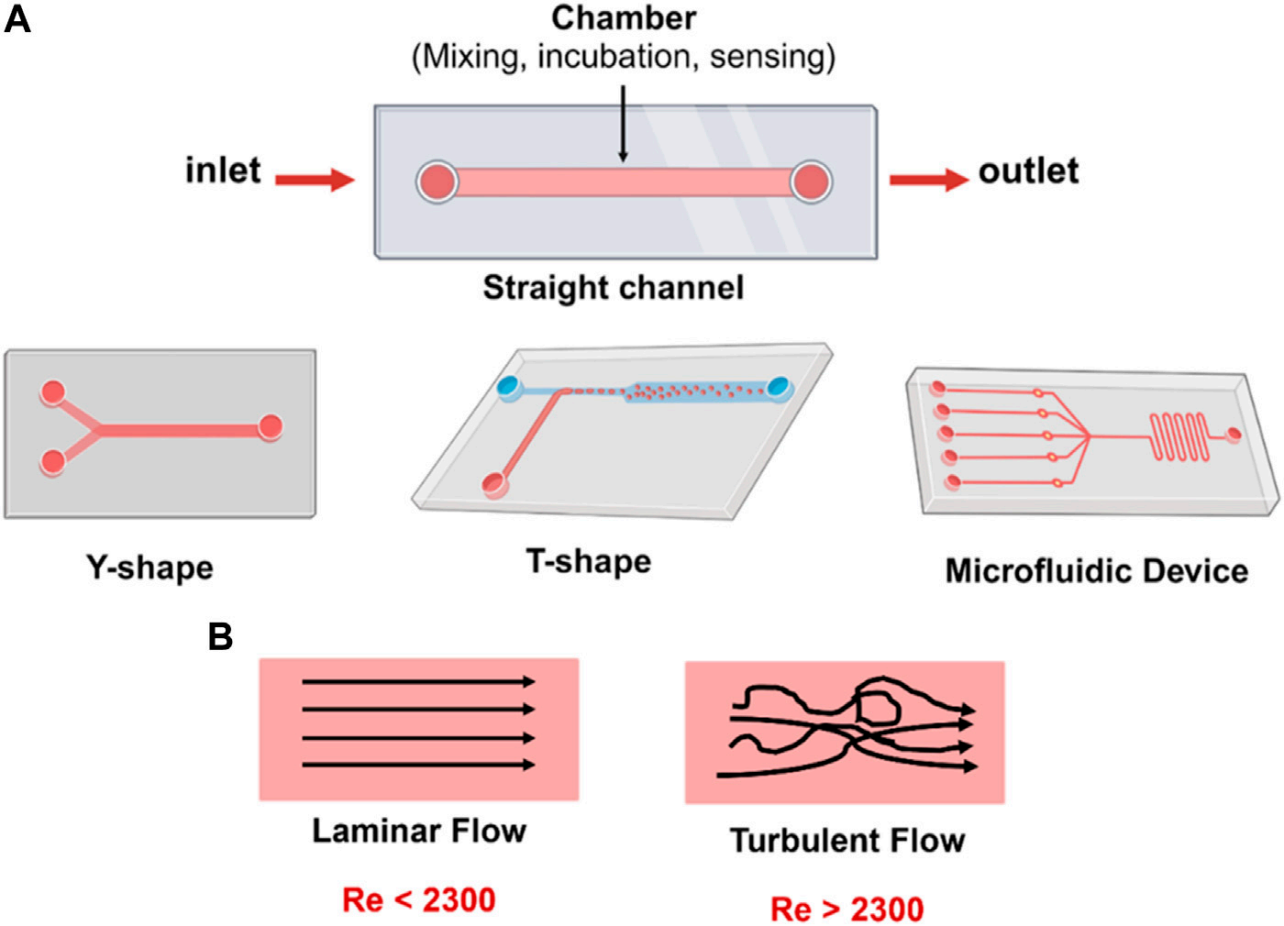
**(A)** Microfluidic chip designs with one or more inlets and one outlet, and different flow geometries; straight channel, Y- and T-shaped channel. **(B)** Reynold’s number (Re) characterizes fluid flow in the microfluidic channels. The Re is classified into either laminar or turbulent. In laminar flow, fluid has distinct streamlines and moves in parallel to the flow direction. In turbulent flow, fluid chaotically flows with no distinct [Created with BioRender.com].

The main components of a microfluidic chip include holes for liquid inlets, outlets, and interconnections to the microchannels or chamber where the reaction occurs (Figure 2A). These inlets, outlets, and channels can have different sizes and shapes based on the chip design and the application. The working process starts by inserting the liquids through the inlet to react or incubate in the microchannels or chamber and discharging the excess liquids through the outlet.

An important principle in microfluidics related to the Reynolds number (Re) must be highlighted to obtain the most benefits from these miniaturized devices and to understand the fluid behavior in these narrow channel systems. The Re is a dimensionless number that describes the ratio of inertial forces to viscous forces and significantly varies for different types of microfluidic chips related to several factors (Rapp and Rapp, 2017; Damiati et al., 2018b). Further, Re can be explained according to the formula Re = $\left(\rho \right.$ VD)/µ, where ρ is fluid density, V is the fluid average velocity, D is microchannel diameter, and µ is the fluid viscosity. If the microchannel of the chip is in non-circular cross-sectional shapes, the D in Re equation is calculated according to the hydraulic diameter (D = 4A/P), where A is the cross-section area, and *p* is the wetted perimeter. However, all the equations indicate the fluid flow behavior in the microchannel (laminar or turbulent). The first is the channel geometry, which is the size and shape. A narrower channel geometry gives a lower Re number. Second, fluid properties such as dynamic viscosity and density, which are part of the Re number equation, have an inverse or direct relationship effect. Third, the flow rate of fluids is related to the fluid velocity. The higher the flow rate, the higher the Re number. Fourth, operation conditions such as pressure and temperature can affect the fluid properties. All these factors play a role in fluid behavior that affects the Re number (Figure 2B). Researchers select the Re number based on the criteria of the application conditions to achieve the required residence time and bio-chemical reaction rates, and mainly prefer to operate under a low Re number to achieve a laminar fluid flow (Shashi Menon and Shashi Menon, 2015; Convery and Gadegaard, 2019; Iakovlev et al., 2022).

A chip can be integrated with different units such as a control driving unit, which consists of a valve and a drive pump like a fluid pressure and injection pump, and a detection unit to amplify and detect signals. Optical and electrochemical detection are widely used in water quality monitoring. Optical methods can use a variety of detection techniques, including Raman spectroscopy, chemiluminescence, and colorimetric methods, which are used to monitor nutrients in water with high efficiency by creating a spectrophotometric device based on microfluidic techniques (Li et al., 2023). The optical method is the easiest and can be coupled with smartphones. The electrochemical method uses amperometric, voltammetric, and potentiometric techniques to convert the chemical signal of the target analyte into an electrical signal via an electrode. The electrochemical method exhibits good detection sensitivity even when miniaturizing the electrode sensing system (Sunaina et al., 2022). The main challenge in using electrochemical detection in water monitoring applications is that real samples are easily polluted and subject to significant interference, which can affect the accuracy of results.

### 2.2 Materials of microfluidic chips

Microfluidic chips can be fabricated from a variety of materials that can control and manipulate the behaviors of the fluids in a chip’s microchannels at the microscale. These materials can forecast the properties of devices chosen for different applications. They can be categorized into inorganic materials, such as silicon, glass, and ceramic; organic materials, such as paper; and polymer materials (Niculescu et al., 2021), such as cyclic olefin copolymer (COC) (trade name “Topas”), cyclic olefin polymer (COP) (trade names “Zeonor” and “Zeonex”), polystyrene (PS), polymethylmetacrylate (PMMA), and polydimethylsiloxane (PDMS).

Glass microfluidic chips are popular in health applications and life sciences because they are reusable, which helps to reduce the cost per use, while most polymeric microfluidic chips are disposable and single-use platforms. Each material has advantages and disadvantages, and the choice of material should be based on the material’s integration degree with the applied application, compatibility with the chemical solutions, and cost-effectiveness, as the cost can be due to the technology used to construct the chip material (Mesquita et al., 2022), not to the material itself. This section highlights the materials used to fabricate microfluidic chips and their advantages and disadvantages.

#### 2.2.1 Inorganic materials

Silicon (Si), glass, and quartz are inorganic materials. Silicon is one of the earliest materials used to fabricate microfluidic chips because of its semiconductor properties, easy modification surface, and the availability of the technology (Nielsen et al., 2020). Inorganic materials possess advantages such as high stability, thermal and electrical conductivity, and solvent compatibility. Nonetheless, inorganic materials have several drawbacks. For example, Si has weak optical properties. Some studies showed that Si-based chips present a deficiency in transmittance in the visible light region but not in the infrared (IR) region. However, glass and quartz are reusable, biocompatible with biological samples, and have good optical properties. As such, the problem of transparent Si materials can be solved by making a hybrid material between glass and Si (Aralekallu et al., 2023).

#### 2.2.2 Organic materials

A diversity of organic polymers are used to design microfluidic chips. Polymers are categorized into thermoplastic polymers, including PMMA, COC, COP, and PS; cured polymers, including PDMS; and volatile solvent polymers, including fluoroplastics. Thermoplastic polymers are rigid due to their structural arrangement, which makes them more resistant to changes in temperature and pressure (Damiati et al., 2022a). Topas and Zeonor demonstrate high efficiencies in their optical characteristics and are compatible with certain organic solvents. PDMS is a hybrid material between silicon and polymer that shows good transparent properties for optical detection methods (Aralekallu et al., 2023). PDMS and PMMA are the main polymers used for environmental monitoring research. PS is used for biological research, such as cell culture. In general, the fabrication processing of polymers is low-cost and more environmentally friendly without the need for a hazardous reagent, and they have good optical transmittance properties and elasticity. The main drawback of polymers is their incompatibility with many organic solvents, which affects device integrity and the reaction or analysis that operates inside a chip. For example, PDMS is highly compatible with commonly used solvents (e.g., chloroform, xylene, and ether) but is susceptible to swelling or chemical attack upon exposure to some acids and bases or nonpolar solvents (e.g., hydrocarbons, toluene, and dichloromethane) (Lee et al., 2003; Vinothkumar et al., 2011). PMMA, PC, and PS are highly compatible with alcohols but not with some organic solvents such as ketones and hydrocarbons. Furthermore, COC is a good choice for microfluidics due to its high resistance to acids and bases (e.g., hydrogen chloride, sulfuric acid, nitric acid, sodium hydroxide, and ammonia) and most organic polar solvents (e.g., acetone, isopropyl alcohol, and methanol). Overall, microfluidic materials are essential parts of the fabrication of devices because they can affect the precision of fluid behavior in microchannels.

#### 2.2.3 Paper materials

Paper-based microfluidic materials represent a good alternative to organic and inorganic materials, especially the latter, which are expensive due to the technology processing techniques. Paper is a sheet containing a compressed hydrophobic/hydrophilic porous membrane that can be made from cellulose fiber or nitrocellulose to control the movement flow of a fluid on the paper via the capillary effect (Jin et al., 2022). This type of material is inspired by the traditional detection technique of paper chromatography (Wu et al., 2022) and has several advantages, such as being easy to modify and functionalize to enhance mechanical strength and conductivity; easily being able to place chemicals on paper; allowing the movement of fluids without needing pump pressure or an external pump; the easy visualization of an analyte’s color development when using colorimetry methods; and low-cost processing because there is no need for harsh fabrication conditions or a closed, clean room as it required for inorganic and organic materials (Ghaseminasab et al., 2023). The main drawback is the low specificity, which is unsuitable for volatile samples, surfactants, and organic solvents. This type of material has garnered more recent interest in the research area of water quality monitoring (Nishat et al., 2021).

## 3 Microfluidics applications in water monitoring

Microfluidic chips show advantages in computability and integration with different detection techniques. This allows researchers to fabricate microfluidic devices with tremendous functionality and properties to be used as detection platforms in applications such as food, water, and medical care diagnostics. There are two main categories of contaminants in water analysis: chemical and biological. Hence, this section highlights recent articles published in the last 5 years on developing microfluidic devices for water quality indices.

### 3.1 LOC applications for detecting chemical contaminants

Chemical contaminants are a huge group of analytes divided into organic and inorganic species. Organic species contain carbon in their primary structure and include chemical oxygen demand (COD), pesticides, volatile organic compounds (VOCs), and organic solvents. Inorganic species do not contain carbon in their primary structure and include nutrients, heavy metals, and anions. Both groups can originate in and enter water resources via human activity, agricultural practices, and industrial waste (Wasewar et al., 2020; Thongam et al., 2021). Some species can also be generated naturally and are important for environmental balance. However, increasing the levels of these contaminants in water can cause eutrophication, so water quality monitoring is essential to human and ecosystem life.

#### 3.1.1 Inorganic contaminants

**FIGURE 3:**
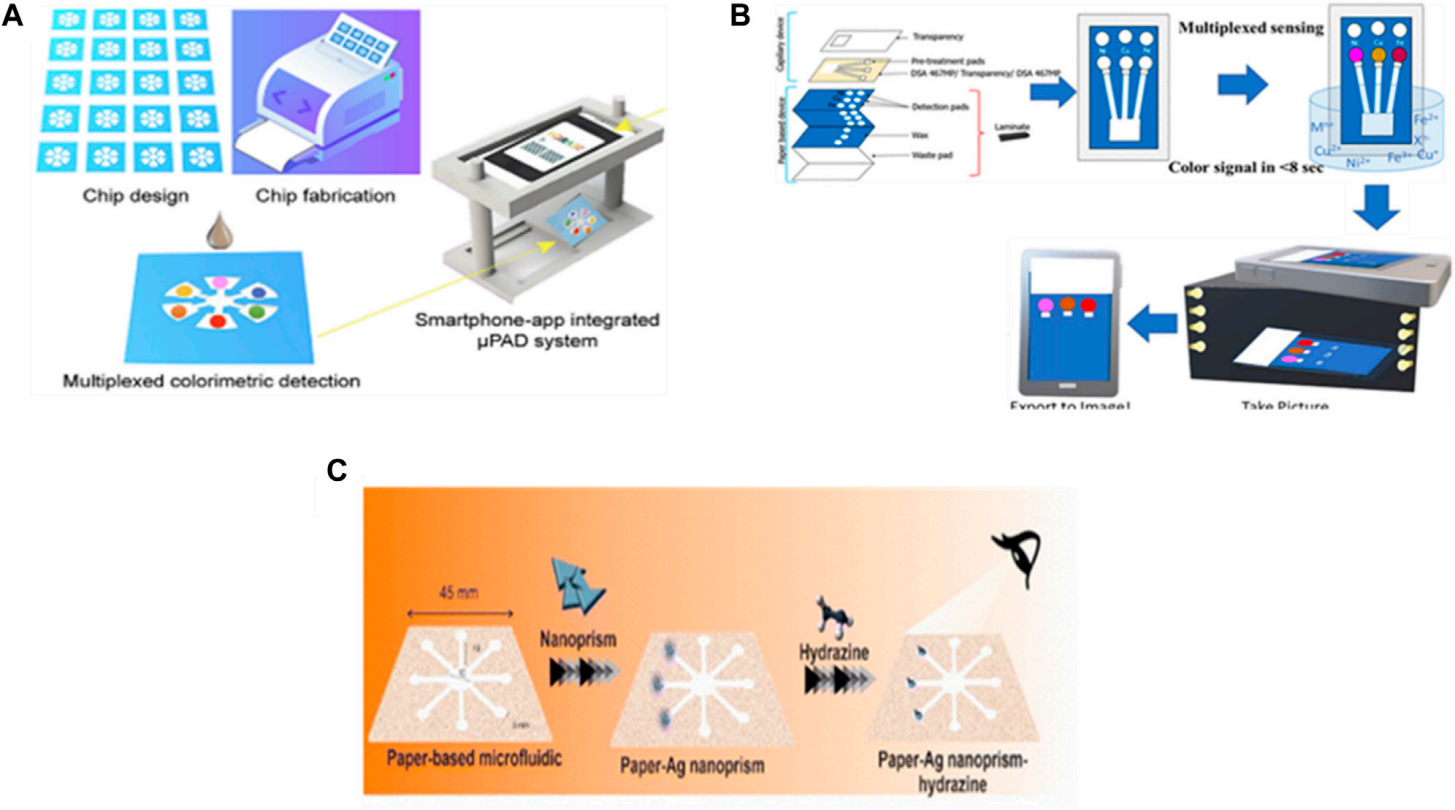
Application of paper-based microfluidic devices for the detection of water quality. **(A)** Development of microfluidic paper-based analytical devices and integration with smartphone-app for simultaneous and fast detection of cross-type multiple water quality parameters including Ni(II), Cu(II), Fe(III), NO^3-^. and pH (Xiong et al., 2022). Reproduced with permission from American Chemical Society. **(B)** Designing of capillary-driven microfluidic device combined with paper for quantification of various metals in water using a smartphone camera and ImageJ software to measure the color intensities (Aryal et al., 2023). Reproduced with permission from American Chemical Society (ACS). **(C)** Microfluidic paper-based colorimetric devices decorated by silver particles for the detection of hydrazine in real samples through UV-visible spectroscopy without the requirement for pre-treatment steps (Ghaseminasab et al., 2023). Copyright 2023, with permission from Royal Society of Chemistry (RSC).

A paper-based analytical device (µ-PAD) is a microfluidic device that is considered one of the lowest-cost and fastest analytical techniques. Xiong et al. (2022) reported on the development of a µ-PAD-based microfluidic device with smartphone app integration for the simultaneous detection of Cu(II), Ni(II), Fe(III), nitrite, and pH (Figure 3A). The device depends on the colorimetric detection method in which the color intensity is proportional to the analyte concentration. The working principle of this system is to measure the color intensity released from a chromogenic reaction that occurs between the reagents, bathocuproine, dimethylglyoxime (DMG), phenanthroline, Griess reagent, and bromothymol blue (BTB), which are added to certain zones in the µ-PAD and the target analytes, Cu(II), Ni(II), Fe(III), nitrite, and pH, respectively. Then, the integration app with the smartphone camera analyzes the captured picture to record the concentrations of the analytes based on the color intensity of each zone. The detection limits and linear ranges of the device were 0.4 ppm and 3.8–400 ppm, 1.9 ppm and 2.9–1,000 ppm, 2.9 ppm and 2.8–500 ppm, 1.1 ppm and 2.3–90 ppm and 5-9 for Cu(II), Ni(II), Fe(III), nitrite, and pH respectively. The recoveries and RSD % ranges were 97.9%–98.4% and 3.12%–4.35%, 98.2%–105.1% and 3.23%–4.34%, 104%–107.6% and 1.38%–2.65%, and 96.7%–106.4% and 3.05%–3.12% for Cu(II), Ni(II), Fe(III), and nitrite respectively. They compared their obtained results of spiked samples with those of inductively coupled plasm–mass spectrometry (ICP–MS) as a standard method for validation were relative standard deviation (RSD) was less than 5%. The smartphone-app-integrated μ-PAD detection system has excellent compatibility and high accuracy and reliability without a significant difference from the ICP–MS method.

In addition, the same type of µ-PAD and reagents were used for the microfluidic-based colorimetric detection of Cu(II), Ni(II), and Fe(III) in water in a study conducted by Aryal et al. (2023). They modified the detection process technique using capillary flow to drive the media in the microfluidic system, without redundantly pipetting the samples into the paper to enhance the color intensity and reduce the detection time to 8 s. Moreover, they used ImageJ software to measure the color intensity of the captured picture by reading the average grey scale. Based on their results, they obtained nearly the same detection limits as the previous study for Cu(II) 0.3 ppm and Ni(II) 2 ppm but a lower one for Fe(III) 1.1 ppm. The quantification limit were 1, 6.67, and 3.67 ppm for Cu(II), Ni(II), and Fe(III) respectively. They achieved good recovery between 80%–110% in more than 90% of real sample matrices from drinking water and rivers with acceptable range of precision and accuracy less than 15% RSD (Figure 3B).

Manbohi and Ahmadi (2022) also successfully executed the quantitative detection of inorganic nutrients, designing µ-PAD microfluidic device integrated with a smartphone app for the simultaneous detection of phosphate, nitrite, and silicate in coastal water. Nutrients are widely used in agriculture for plant growth. They can be carried to waterbodies via rainfall, so simultaneously monitoring the nutrients in waterbodies is crucial to avoid the overgrowth of aquatic plants and eutrophication. The researchers' device can be used for onsite monitoring and is based on the principle of colorimetric detection, which relates the intensities of RGB components in the captured picture to the concentrations of the analytes. The detection limits were 1.52, 0.61, and 3.74 μg/L for phosphate, nitrite, and silicate, respectively. The linear ranges were between 5 and 100 μg/L for phosphate (*R* ^2^ = 0.9909), 5 and 100 μg/L for nitrite (*R* ^2^ = 0.9819), and 10 and 600 μg/L for silicate (*R* ^2^ = 0.9933). Their sensor exhibits good agreement compared with a spectrophotometric method using a real coastal water sample, where the recovery percentages were 92%–108%, 95%–105%, and 94%–103% for phosphate, nitrite, and silicate, respectively. Furthermore, it shows advantages in the total analysis time less than 6 min and generating real-time maps and sharing the results via a network.

Hydrazine (N_2_H_4_) is an inorganic compound widely used in industries and has a carcinogenic effect on human health. Humans may be exposed to it by drinking contaminated water. Hence (Ghaseminasab et al., 2023), developed a portable tool for detecting hydrazine that does not require pretreatment or reagents and is based on a microfluidic paper-based colorimetric device (µ-PCD). Furthermore, it requires the naked-eye colorimetric monitoring of N_2_H_4_. The designed µ-PCD is fabricated from paraffin as it is an inexpensive material and modified with silver nanomaterials (silver nanoprisms (AgNPrs)), silver nanowires (AgNWs), and silver citrate (AgCit)). In this portable device, the AgNWs and AgNPrs show outstanding colorimetric results for N_2_H_4_ detection among a linear range of 0.08–6 M and 0.02–5 M respectively, as well as lower limit of detection 800 μM and 200 µM were stable for 90 and 60 days, respectively (Figure 3C). Generally, the reported studies that depended on paper-based microfluidic chip materials showed the simplicity of the detection procedure and could identify the contaminant primarily with the naked eye. However, to know the actual concentration, they integrated with a mobile app that needed an efficient algorithm.

**FIGURE 4:**
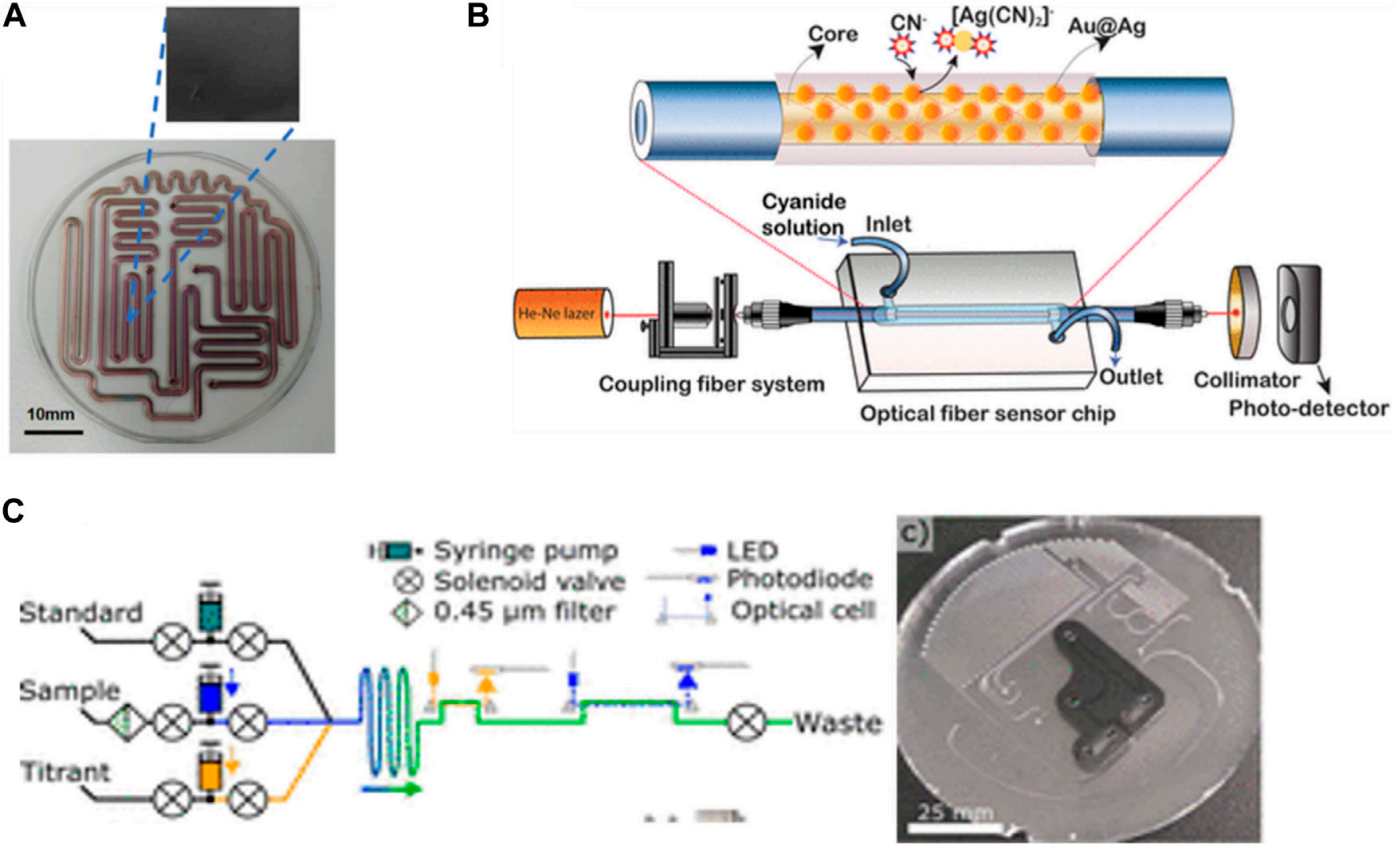
**(A)** Fabrication of a glass microfluidic device with a diamond-like carbon-coated channel surface to measure residual chlorine in tap water Via N,N-diethyl-p-phenylenediamine (DPD) method (Tazawa et al., 2023). Copyright 2023, with permission from Springer Nature. **(B)** Fabrication of a fiber optical microfluidic sensor chip with PDMS and Au@Ag NPs to detect cyanide ions (CN) in real-time by monitoring changes in the fiber cladding refractive index (Phuong et al., 2023). Reproduced with permission from American Chemical Society (ACS). **(C)** *In situ* analyzer for seawater total alkalinity and the design of the PMMA microfluidic chip with channels and inlaid optical cells (Sonnichsen et al., 2023). Reproduced with permission from Reproduced with permission from American Chemical Society (ACS).

Microfluidic devices can be made from different materials to enhance the properties of detections. Tazawa et al. (2023) introduced a microfluidic device using a glass-molding process for the onsite measurement of residual chlorine in tap water. The chip was fabricated from a glass substrate and then coated with diamond-like carbon (DLC) to prevent the mineral’s adsorption effect (Figure 4A). The device can detect residual chlorine in the range of 0.1–1 ppm. In addition (Phuong et al., 2023), fabricated a sensitive device for cyanide monitoring in water based on the hybrid coating nanomaterial of Au_core_–Ag_shell_. The fiber optical sensor was clad with cyanide and formed a cyano–metal complex that led to a change in the refractive index of the localized surface plasmon resonance peak (Figure 4B). This method is more environmentally friendly due to the low toxicity of the nanomaterial compared with the reagent used in the traditional detection method. They reported the lowest detection method was 8 × 10^−11^ M over the concentration range of CN^−^ between 0–150 µM. In general, microfluidic chips with different materials and integration methods show a significant advantage in detecting various chemical contaminants that can pose serious risks to human health and the environment.

Meanwhile, microfluidic devices have made a breakthrough in the field of *in situ* sensors due to the low consumption volume of samples and reagents, which can lead to analyzing a maximum number of samples. Some researchers have tried to build more advanced *in situ* sensors automated without human intervention. Sonnichsen et al. (2023) proposed an automated LOC device for analyzing total alkalinity *in situ* in seawater (Figure 4C). The developed microfluidic device enabled fluid mixing, chemical reactions, and optical detection on a single platform. The device demonstrated satisfactory results of the analysis in the 5–25°C range, and a 1 L acid/indicator bag with a 500 mL standard bag was enough to analyze 532 samples and 62 standard measurements due to the advantage of low reagent consumption in microfluidics. Moreover, this device provided instant and permanent results of the seawater’s total alkalinity. Total alkalinity is an efficiency indicator of ocean alkalinity enhancement (OAE) for the capture and removal of CO_2_ from the air and dissolved in the ocean by being converted to bicarbonate, which can reduce the effect of CO_2_ and decrease the crisis of increasing temperatures and climate change impacts. However, the main problem is if the ocean is acidified due to saturation with bicarbonate, which can affect the ability to capture CO_2,_ so monitoring the total alkalinity is very important to take quick action to reduce the acidification influence by enhancing the ocean’s alkalinity.

Another study by Morgan et al. (2022) reported on a fully automated phosphate analyzer based on an inlaid microfluidics absorbance cell. The microfluidic chip fabricated from polymethyl methacrylate (PMMA) consists of a syringe pump and solenoid valves to aid in automated fluid control. Their developed device achieved a 15.2 nM detection limit and a 50.8 nM quantification limit over a dynamic range of 0.2–10 µM. Also, the sensor shows high *in situ* measurements underwater with (<1.5%) RSD. However, these types of automated sensors need highly expert scientists to build a good architecture sensor. Both studies presented excellent models with high efficiencies.

#### 3.1.2 Organic contaminants

**FIGURE 5:**
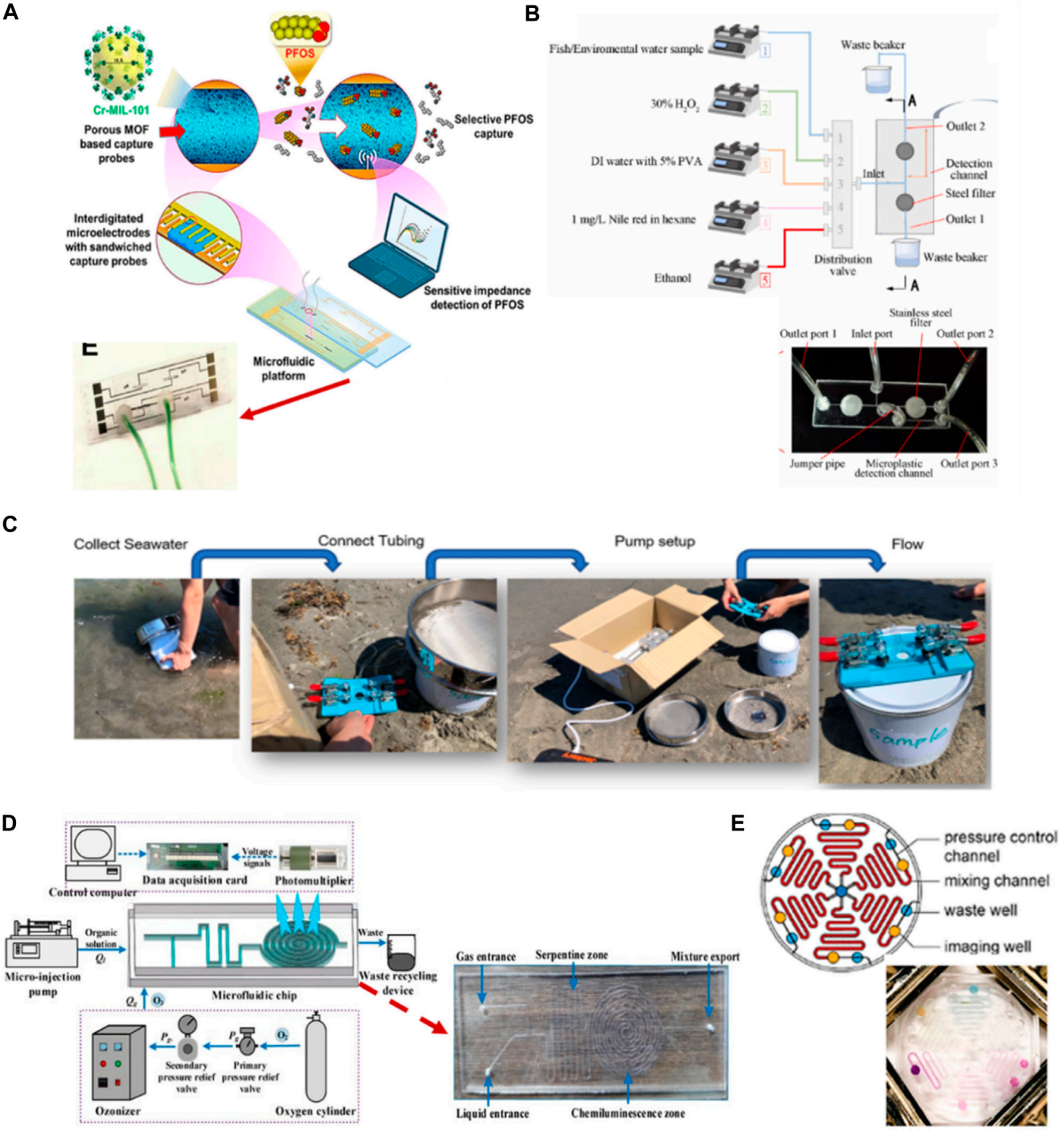
**(A)** Development of nanoporous, interdigitated electrodes on a standard glass slide to develop a microfluidic flow-through platform for PFOS detection using specific affinity-based interactions between MOF-based receptor and PFOS (Cheng et al., 2020). Reproduced with permission from American Chemical Society (ACS). **(B)** Fabricated two-layer PMMA-based microfluidic chip for microplastic analysis allowed sample digestion, filtration and counting processes within single platform with the preprogrammed sequence. Fluorescence microscope and video processing software were used to quantify microplastic in river water sediment and fish gastrointestinal tract contents (Zhang et al., 2023). Reproduced with permission from Elsevier. **(C)** A PDMS microfluidic device featuring sieve-like structures designed specifically for on-site, label-free identification of small-sized microplastics in seawater (Gong et al., 2023). Copyright 2023, with permission from Springer Nature. **(D)** Development of micro-fine bubbles under T-type flow-focused PDMS microfluidic chip combined with ozone chemiluminescence for COD detection system (Li et al., 2022). Copyright 2021, MDPI. **(E)** Design of multiplexed PMMA microfluidic device for detection of COD, ammonia, nitrogen, nickel, chromium and phosphate. Color intensities were quantified on the chip using cell phone at different concentrations (Jiang et al., 2020). Reproduced with permission from American Chemical Society (ACS).

The advantages of using microfluidic chips in the detection of inorganic contaminants in water can be applied to the detection of organic contaminants. Per- and poly-fluoroalkyl substances (PFASs) and microplastics are new analytes that the EPA considers toxic to human health. PFASs are a large group of organic substances, including perfluorooctanesulfonate (PFOS). A recent study showed that approximately 45% of PFASs detected in US drinking water and the substances humans are most exposed to are PFOA and PFOS (Smalling et al., 2023). Rapid detection sensors are necessary to protect human health. The microfluidics technique could keep up with new types of contaminants. Cheng et al. (2020) present microfluidic impedance sensors based on a metal–organic framework (MOF) for PFOS analysis (Figure 5A). The principle of the sensor is based on the design of a receptor probe made from the MOF along with a microelectrode in a microfluidic channel through which the fluid passes to capture PFOSs. They present the advantages of the flow technique to overcome the diffusion resistance of the solution and allow for rapid measurement with a detection limit 0.5 ng/L. Zhang et al. (2023) produced a microfluidic device for integrated sample processing and counting for microplastic analysis. The device shows simplicity in analysis and reduces costs compared with the traditional technique. The method is semi-automated to perform the digestion, filtration, and staining of microplastics in the river water sample inside the microfluidic chip, which is fabricated from a double layer of PMMA. The microplastics are counted and detected with a fluorescence microscope, and the results are then processed with a software video (Figure 5B). In addition (Gong et al., 2023), developed a model that combined a microfluidic chip and machine learning to identify tiny-sized (smaller than 50 µm) microplastics in seawater. The chip made from PDMS was used to trap a tiny pristine microplastic particle to overcome the frequent overlapping of peaks encountered in direct analysis using Raman spectroscopy (Figure 5C). Moreover, using a microfluidic chip can trap a single particle and increase the accuracy of detection and identification with real samples. This developed model is much more complicated when combined with different machine learning models compared with the previous study, but it can save a lot of time in identifying new types of microplastics.

Some important organic pollutants need rapid treatment action due to their effects. COD is an important water index that indicates the degree of organic species in water that can compete with organisms for oxygen and the efficiency of plant treatment. Dichromate or potassium permanganate digestion is the traditional detection method used in the laboratory. It harms the environment due to the reagent’s toxicity and the volume used for each sample.

Li et al. (2022) present a COD detection technology using a t-structure microfluidic chip based on the ozone chemiluminescence detection method principle, offering sensitivity, rapidity, and no formation of byproduct pollutants (Figure 5D). This method shows COD detection results consistent with those obtained using the traditional potassium dichromate method with an average deviation of less than 5%. In addition (Jiang et al., 2020), developed a multiplexed chip for simultaneously detecting five parameters for monitoring water quality, including COD. The method is based on the color reduction of permanganate from purple to green, followed by analyzing the color intensity of the captured picture with a smartphone using MATLAB (Figure 5E). This device represents a quantification range for COD-low concentration (1–50) mg/L and COD-high concentration (50–250) mg/L. The percentage error of the analysis using the method device compared with a standard method was 3% for the sample from river and 1% for the sample from industrial waste. Compared with the previous study, this chip demonstrates the simplicity of fabricating the device, and the small amount of reagent required reduces the amount of chemical waste produced via the reactions.

**FIGURE 6:**
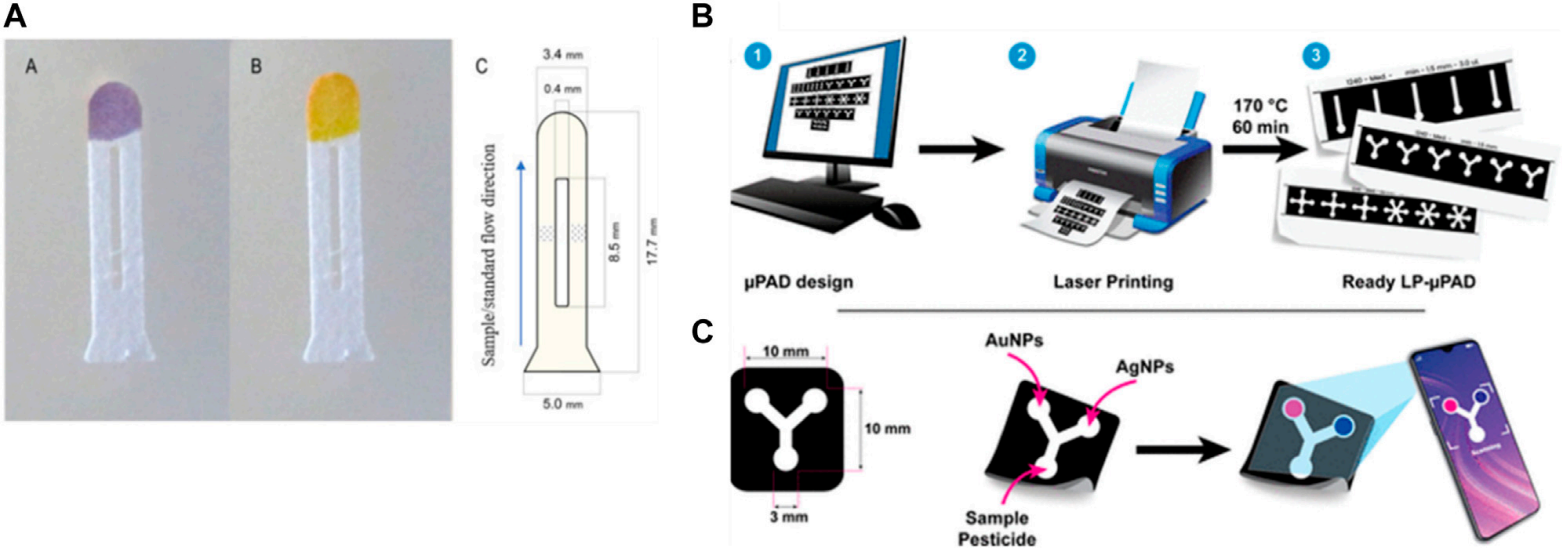
**(A)** Paper-based microfluidic for organophosphorus pesticides detection. The device consists of three separate zones, one for sampling and two transport channels separated by a gap where the acetylcholinesterase and acetylcholine chloride solutions are deposited in each one, and a detection zone containing the pH indicator. A purple color and yellow color were produced in the presence and absence of the pesticide, respectively (Fernández-Ramos et al., 2020). Copyright 2020, Elsevier. **(B, C)** Designing, printing, and baking the printed paper to produce paper-based microfluidic device for Atrazine detection in water. Silver and gold nanoparticles decorated the sensing area and in the presence of the pesticide a color developed. The color intensity was measured by smartphone camera and ImageJ at different concentrations of atrazine pesticide (Moulahoum, 2023). Copyright 2023, American Chemical Society (ACS).

Pesticides are also considered a type of organic contamination. They are introduced into waterbodies via agricultural and non-agricultural practices, and some types pose cariogenic risks to human health. Fernández-Ramos et al. (2020) developed a microfluidic paper based on the optical detection of organophosphate pesticides and carbamate. The determination process was based on inhibiting the catalytic activity of acetylcholine esterase (AChE) in the presence of organophosphorus and carbamate, which can be hydrolyzed with acetylcholine chloride (AChCl), leading to color changes (Figure 6A). Then, ImageJ software was used to calculate the intensity of the captured picture with a camera. The microfluidic chip used was a double µ-PAD, which helped separate the channel reagent path added for the detection process to avoid the direct contact of the substrate with the enzyme before adding the samples. This device showed a good recovery percentage with real samples between 97.7% and 102.3% with a detection limit of 0.24 μg/L for carbaryl and 2.00 for chlorpyrifos. Also, with reproducibility between 4.2%–5.5%. In addition, Moulahoum (2023) reported a method for detecting atrazine, a pesticide used for crops, based on fabricating a biosensor from a laser-printed µ-PAD chip in a Y shape using gold and silver nanoparticles as reagents for colorimetric detection (Figure 6B). The µ-PAD chip was fabricated useing a laser-printed to reduce the devices cost compared to fabrication with wax or inkjet printer. The color sensing was based on the aggregation of metallic nanoparticles due to the ligand exchange with the pesticide. The captured picture of the color signal was analyzed using ImageJ software. The detection limit was 3.5 µM using AgNPs and 10.9 µM using AuNPs. The recovery percentage of the spiked water samples with atrazine was in good agreement with the spiked concentration between 100.2% and 103.4% for AgNPs-based sensor and 117%–122% for AuNPs-based sensor. Both studies show the simplicity of the devices and detection. Nevertheless, the pesticides consist of different species types that can interfere. Table 1 shows different microfluidic platforms to detect chemical contaminants in water using various detection methods.

**TABLE 1:** Summary of recently reported microfluidics in monitoring water quality.

Analytes	Materials of microfluidics	Detection methods	Limit of detection	References
Cu(II), Ni(II), Fe(III), Nitrite, and pH	µ-PAD	Colorimetric	0.4 ppm - Cu(II)	Xiong et al. (2022)
			1.9 ppm - Ni(II)	
			2.9 ppm - Fe(III)	
			1.1 ppm - nitrite	
Cu(II), Ni(II), Fe(III)	µ-PAD	Colorimetric	0.3 ppm - Cu(II)	Aryal et al. (2023)
			2 ppm - Ni(II)	
			1.1 ppm - Fe(III)	
Phosphate, Nitrite, and Silicate	µ-PAD	Colorimetric	1.52 μg/L -phosphate	Manbohi and Ahmadi (2022)
			0.61 μg/L–nitrite	
			3.74 μg/L - silicate	
Hydrazine	µ-PCD	Colorimetric	800 µM -AgNWs 200 µM -AgNPrs	Ghaseminasab et al. (2023)
Residual Chlorine	Glass	Optical	0.1–1 ppm	Tazawa et al. (2023)
Cyanide	PDMS	Optical	8 × 10^−11^ M	Phuong et al. (2023)
Total alkalinity	PMMA	Optical	-	Sonnichsen et al. (2023)
Phosphate	PMMA	Electrochemical	15.2 nM	Morgan et al. (2022)
PFOS	Glass	Electrochemical	0.5 ng/L	Cheng et al. (2020)
Microplastic	PMMA	Optical	-	Zhang et al. (2023)
Microplastic	PDMS	Optical	-	Gong et al. (2023)
COD	PDMS	chemiluminescence	-	Li et al. (2022)
COD	µ-PAD	Colorimetric	COD-low (1–50) mg/L	Jiang et al. (2020)
			COD-high (50–250) mg/L	
Organophosphate and Carbamate	µ-PAD	Colorimetric	0.24 μg/L - carbaryl 2.00 μg/L - chlorpyrifos	Fernández-Ramos et al. (2020)
Atrazine	µ-PAD	Colorimetric	3.5 µM - AgNPs	Moulahoum (2023)
			10.9 µM - AuNPs	

### 3.2 LOC applications for detection of biological contaminants

The second group of water contaminants comprises biological species such as pathogens and bacteria. This is the worst type of contaminant because it can infect the human immune system at trace levels without needing an accumulation period until the impact appears like chemical contamination. The main challenge with the traditional methods for detecting biological contamination is the long time required for the occupation and growth of bacteria.

**FIGURE 7:**
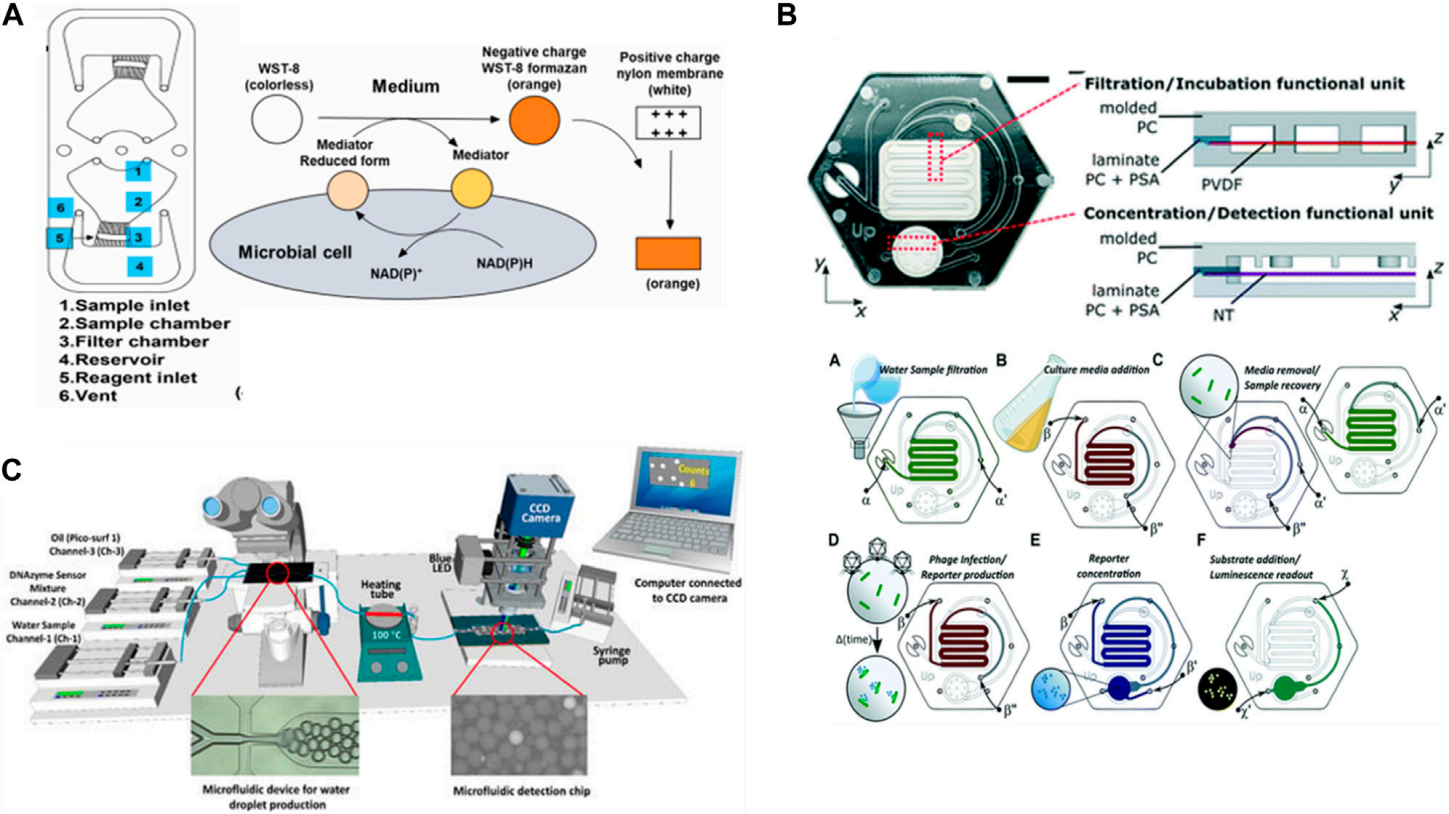
**(A)** Fabrication of a nylon membrane hybrid portable centrifugal microfluidic device to detect live bacteria in water. WST-8 was used for colorimetric detection of microbial metabolism and data analyzed visually or recorded with a smartphone camera and then processed by ImageJ software (Chang et al., 2023). Reproduced with permission from Elsevier. **(B)** Phage-based microfluidic platform made with polycarbonate enclosures and polyvinylidene difluoride membrane, nitrocellulose membrane, hydrophobic membrane, for filtering *Escherichia coli* cells from water samples, for concentrating reporter, and for venting the device channels, respectively (Alonzo et al., 2022). Reproduced with permission from American Chemical Society (ACS). **(C)** The developed system composed of two microfluidic devices. The first glass microfluidic chip to produce water droplets and to isolated *Escherichia coli* into individual droplets containing a DNAzyme mixture. After bacterial cell lysis by heating, the droplets passed through the second PDMS/PMMA microfluidic device for collecting the fluorescence signal from the DNAzyme sensor of single *Escherichia coli* lysed encapsulated inside the droplets (Rauf et al., 2022). Copyright 2022, MDPI.

Microfluidic devices play crucial roles in the detection of biospecies. Chang et al. (2023) used integration centrifugal microfluidics with nylon filter membranes to rapidly detect live bacteria in water. The method concept was to concentrate the bacteria via a centrifugation system and then occupy them with water-soluble tetrazolium-8 (WST-8), which was used for the colorimetric detection of the bacterial metabolism so changes in the nylon membrane color were related to the concentration of live bacteria such as *E. coli (E. coli)*. The chip was fabricated from PMMA, and the detection limit was 10^2^ CFU/mL in the range of 10^2^–10^5^ CFU/mL during the 3 h of the detection process (Figure 7A). Additionally (Alonzo et al., 2022), identified a method using a microfluidic device and a prototype instrument based on phage-bioluminescence assay for detecting *E. coli*. Their method achieved highly sensitive detection of single-cell levels of *E. coli* in a shorter time than that required for traditional methods. They used a phage-based assay in the microfluidic platforms with the filtration membrane to identify the bacteria in drinking water samples (Figure 7B). In addition (Rauf et al., 2022), developed a method using microfluidics and computer software based on DNAzyme to isolate and detect *E. coli* in water samples. In this method, *E. coli* was trapped in single droplets containing a DNAzyme mixture. Then, the water droplets were heated to lyse the bacteria encapsulated inside. The DNAzyme mixture reacted with the substrate present in the *E. coli*, which led to the dissociation of the fluorophore–quencher pair in the DNAzyme and emitted fluorescence signals that indicated the presence of *E. coli* in the droplets. A smart software computer was developed to help count the fluorescence droplets and distinguish them from non-fluorescence droplets. Furthermore, this processing method demonstrated the highly specific detection of *E. coli* in the presence of other bacterium types (Figure 7C). These examples show that the sensitivity of the detection of *E. coli* using different integration techniques with a microfluidic chip can be according to the sample’s matrix complexity.

Furthermore, (Jin et al., 2023) developed a microfluidic biosensor based on a colorimetric method using finger-driven mixing and nuclear track membrane filtration for the detection of *Salmonella*. They used immune Au@Pt nanoparticles to label the bacteria. The bacteria and immune Au@Pt NPs were pipetted into the mixing chamber to form conjugates and then flowed into the microfilter membrane, where the H_2_O_2_-TMP was pipetted to catalyze the conjugates. After that, they applied ImageJ to calculate the color intensity due to the reaction and detected *Salmonella* using colorimetric methods in 25 min at levels as low as 168 CFU/mL. This new and attractive sensor technology shows great potential in pathogen contamination detection. Many researchers have presented their work in this field, which opens the possibility of discovering new paths for other types of bacteria.

## 4 Challenges

Many researchers have tried to fabricate LOC sensors with a low cost and high sensitivity; however, further clarifications and trials are needed. Water is a big category that can refer to drinking water, such as bottled and unbottled water, or environmental water, such as lakes, effluents, and discharge, which is the most complex matrix compared with drinking water. This complexity of the matrix contributes to one of the main challenges of LOC sensors, which is integrating the pretreatment step into the LOC device. Most methods conduct a pretreatment stage before injection into a microfluidic device or integrating the LOC device with another device. The second main challenge is the integration of smart systems and interferences. Water security is an important global issue. Onsite sensors are necessary in countries with a deficit in permanent laboratories and expensive instruments for monitoring water indices. However, onsite sensors suffer from the complexity of the devices' app algorithms or the stability of the device architectures. Furthermore, most simple detection methods are based on colorimetric techniques, which can cause overlapping specificity or false results, so it is crucial to optimize the right conditions. Building knowledge from different articles regarding different contaminants can help identify the procedure for the best detection conditions.

## 5 Conclusion

Microfluidics is an advanced technology in the field of detection and sensors due to the miniaturization systems that can control the fluid’s movements through the microchannels and chambers. Microfluidic devices can be fabricated from different types of materials, and each has properties that depend on its purpose or application. Microfluidic devices possess tremendous capabilities with different techniques and systems for the detection of various chemical and biological contaminants in water, as proven by their use in research for detecting nutrients, metals, total alkalinity, organic compounds, and pathogens such as *E. coli* and *Salmonella*. This field of research and applications still faces significant challenges. Building knowledge of the best practices will open the doors for future work in the field of microfluidics sensors.
